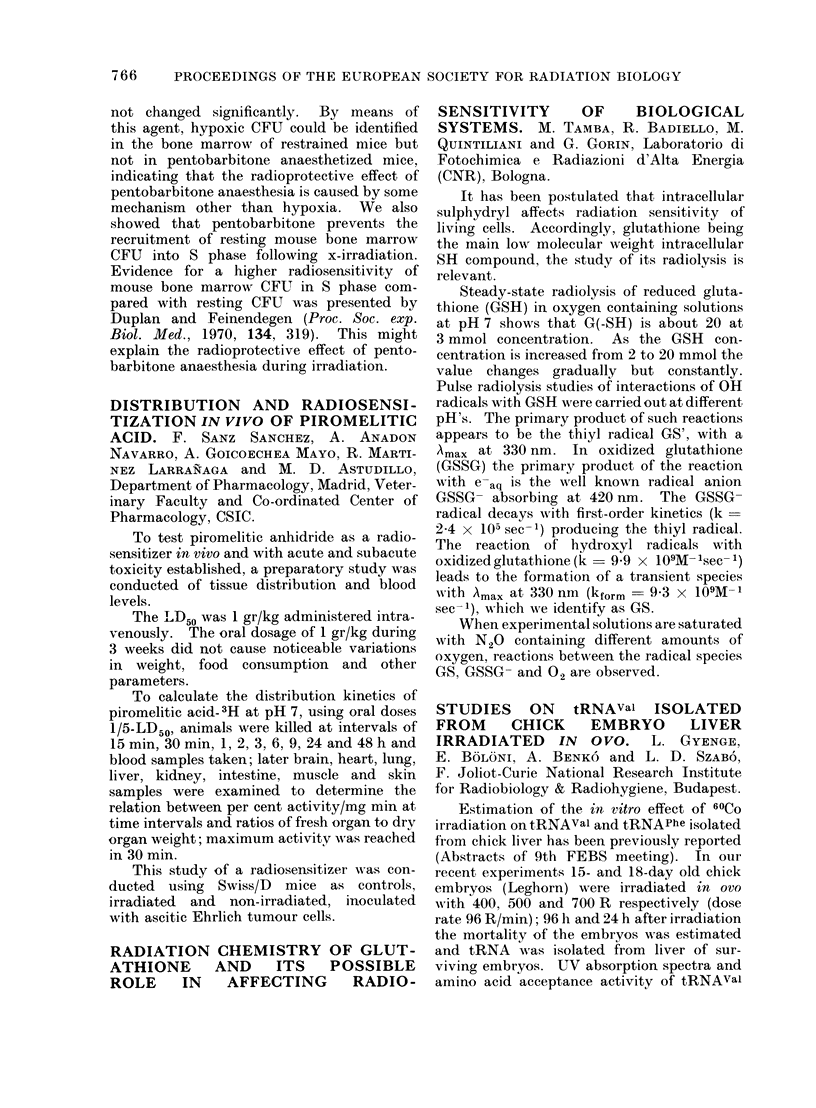# Proceedings: Distribution and radiosensitization in vivo piromelitic acid.

**DOI:** 10.1038/bjc.1975.338

**Published:** 1975-12

**Authors:** F. S. Sanchez, A. A. Navarro, A. G. Mayo, R. M. Larrañaga, M. D. Astudillo


**DISTRIBUTION AND RADIOSENSITIZATION *IN VIVO* OF PIROMELITIC ACID.** F. SANZ SANCHEZ, A. ANADON NAVARRO, A. GOICOECHEA MAYO, R. MARTINEZ LARRAÑAGA and M. D. ASTUDILLO, Department of Pharmacology, Madrid, Veterinary Faculty and Co-ordinated Center of Pharmacology, CSIC.

To test piromelitic anhidride as a radiosensitizer *in vivo* and with acute and subacute toxicity established, a preparatory study was conducted of tissue distribution and blood levels.

The $LD_{50}$ was 1 gr/kg administered intravenously. The oral dosage of 1 gr/kg during 3 weeks did not cause noticeable variations in weight, food consumption and other parameters.

To calculate the distribution kinetics of piromelitic acid-$^3$H at pH 7, using oral doses 1/5-$LD_{50}$, animals were killed at intervals of 15 min, 30 min, 1, 2, 3, 6, 9, 24 and 48 h and blood samples taken; later brain, heart, lung, liver, kidney, intestine, muscle and skin samples were examined to determine the relation between per cent activity/mg min at time intervals and ratios of fresh organ to dry organ weight; maximum activity was reached in 30 min.

This study of a radiosensitizer was conducted using Swiss/D mice as controls, irradiated and non-irradiated, inoculated with ascitic Ehrlich tumour cells.